# Transtibial Anterior Cruciate Ligament Reconstruction Using a Synthetic Graft Combined With Remnant Repair: A Hybrid Technique

**DOI:** 10.1002/atn2.70160

**Published:** 2026-07-09

**Authors:** Peng Wang, Yirui Han, Weibo Yin, Yufei Zhang, Weiliang Zhong

**Affiliations:** ^1^ Department of Joint and Sports Medicine First Affiliated Hospital of Dalian Medical University Dalian China; ^2^ Key Laboratory of Molecular Mechanism for Repair and Remodeling of Orthopaedic Diseases Dalian Liaoning Province China

## Abstract

The purpose of this technical note is to describe a hybrid arthroscopic technique that combines anterior cruciate ligament reconstruction using a synthetic graft with concomitant remnant repair. Following diagnostic arthroscopy and assessment of remnant quality, tibial and femoral tunnels are created using a transtibial approach with attention to graft isometry. Anterior cruciate ligament remnant fibers are selectively preserved and repaired using a cinch‐loop suture followed by an additional encircling stitch to form a sleeve‐like construct. A synthetic ligament is then passed through the prepared tunnels, and the repaired remnant is cofixed with the synthetic graft on the femoral side, allowing the remnant to envelop the graft. This technique provides immediate mechanical stability while preserving vascularized native tissue and neuromechanical elements that may support biological integration and proprioceptive preservation.

## Graphical abstract

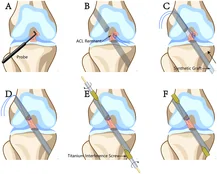

**VIDEO 1:** Arthroscopic illustration of transtibial anterior cruciate ligament reconstruction using a synthetic ligament combined with concomitant remnant repair. The video illustrates diagnostic arthroscopy with assessment of anterior cruciate ligament remnant quality and selective preservation of viable remnant fibers. Transtibial creation of the tibial and femoral tunnels with attention to near‐isometric graft positioning is shown, followed by remnant repair using a cinch‐loop suture and an encircling stitch to create a sleeve‐like construct. Passage of the synthetic ligament through the repaired remnant sleeve is shown, with delayed tensioning of the remnant sutures to facilitate controlled graft insertion. Femoral‐side cofixation of the synthetic ligament and remnant sutures and tibial fixation of the synthetic ligament are illustrated. Final dynamic and arthroscopic assessments confirm complete graft containment within the remnant sleeve, appropriate tension, restored anterior knee stability, and absence of notch impingement or cyclops formation. Video content can be viewed at https://doi.org/10.1002/atn2.70160.

Synthetic ligaments have regained attention in anterior cruciate ligament (ACL) reconstruction owing to their immediate mechanical strength, avoidance of donor‐site morbidity, and suitability for early rehabilitation.^1^, ^2^ However, despite these mechanical benefits, synthetic grafts remain biologically limited. Their tendency toward poor synovialization and insufficient vascular ingrowth restricts long‐term incorporation and may expose the graft to inflammatory reactions or tunnel dilation.^3^, ^4^

ACL remnant‐preserving and remnant‐repair techniques have attracted increasing interest because native ACL fibers contain biological and neuromechanical elements that may support healing and proprioceptive recovery.^5^, ^6^ Retention of remnant tissue may facilitate revascularization, promote synovial coverage, and contribute to proprioceptive function.^7^, ^8^ However, despite these biological advantages, remnant repair alone may be mechanically insufficient.^9^, ^10^ Repaired fibers may not reliably withstand early postoperative loads, particularly in high‐demand or athletic individuals. In addition, excessive or disorganized remnant tissue may predispose patients to cyclops lesions or impingement,^11^, ^12^ while inconsistent remnant quality limits the reproducibility of the technique. These challenges highlight the need for a reconstructive strategy that preserves the biological value of the remnant without compromising mechanical stability.

To address this need, we describe a hybrid technique that integrates synthetic ligament reconstruction with concomitant ACL remnant repair. By allowing the remnant to function as a biologically active envelope over the synthetic graft, this approach aims to enhance synovialization, support graft incorporation, and potentially improve proprioceptive recovery without compromising mechanical stability.

## SURGICAL TECHNIQUE

**FIGURE 1:**
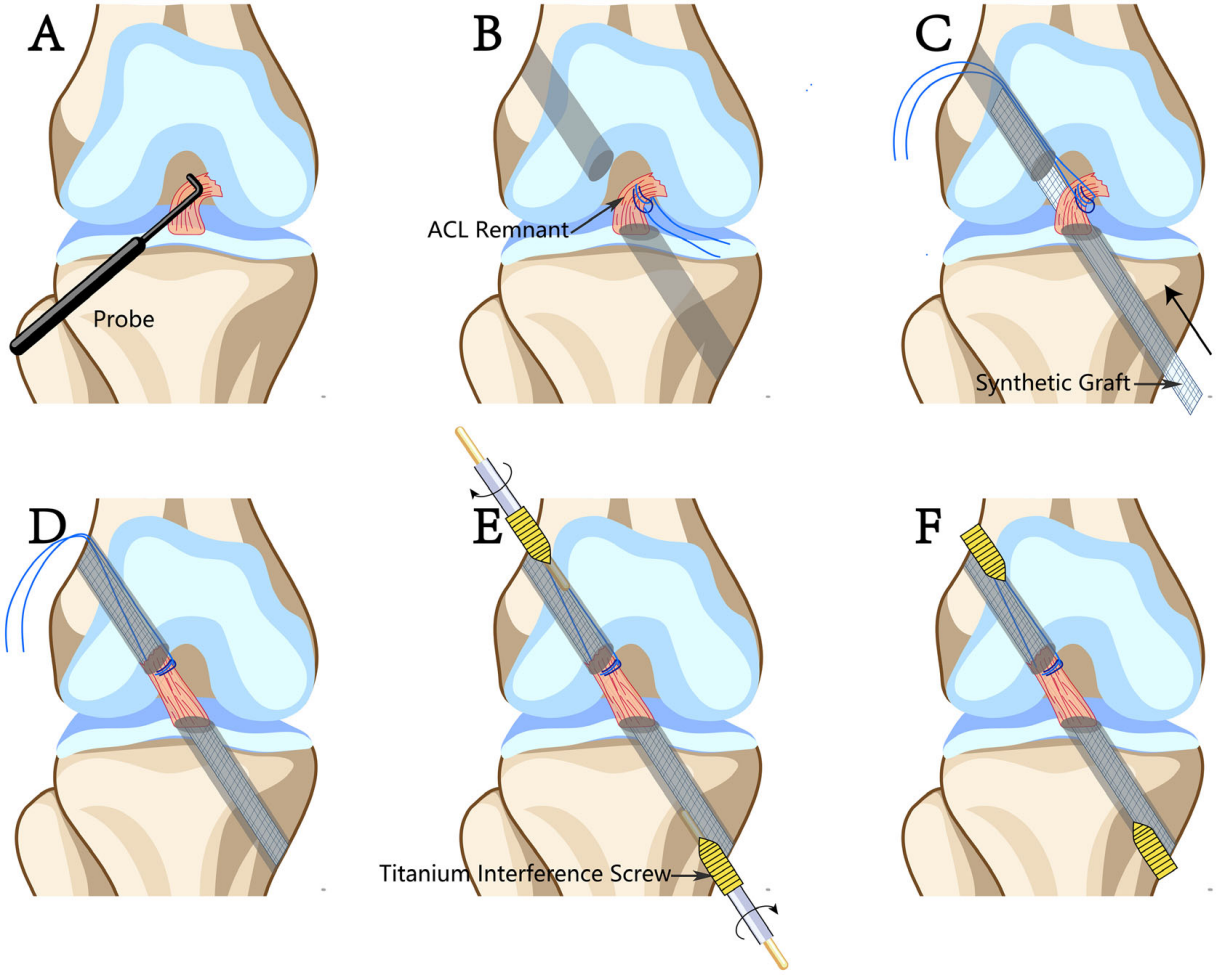
Schematic illustration of the hybrid ACL reconstruction technique, in which a synthetic graft is combined with concomitant remnant repair to form a sleeve‐like construct. (A) Arthroscopic assessment of the ACL remnant. (B) Remnant repair using a cinch‐loop and encircling suture configuration. (C) Sequential advancement of the remnant sutures and the synthetic ligament into the femoral tunnel. (D) Gradual tensioning of the remnant sutures to allow sleeve‐like coverage of the synthetic ligament. (E) Femoral‐side interference screw fixation of the synthetic graft together with the remnant sutures, followed by tibial fixation of the synthetic graft. (F) Final construct showing complete coverage of the synthetic ligament by the repaired remnant. (ACL, anterior cruciate ligament.)

Our technique is shown in detail in Video 1 and is described in the following steps (Figure 1).

### Patient Positioning and Preparation

The patient is placed supine under general anesthesia with a pneumatic tourniquet applied to the proximal thigh. The operative knee is positioned at 90° of flexion in a padded leg holder. Standard arthroscopic instruments and tunnel guides are prepared.

### Portal Placement

Standard anterolateral and anteromedial portals are established with the knee flexed to 90°. An accessory medial portal is created as needed to facilitate remnant suturing.

### Step 1: Remnant Evaluation and Biological Preparation

**FIGURE 2:**
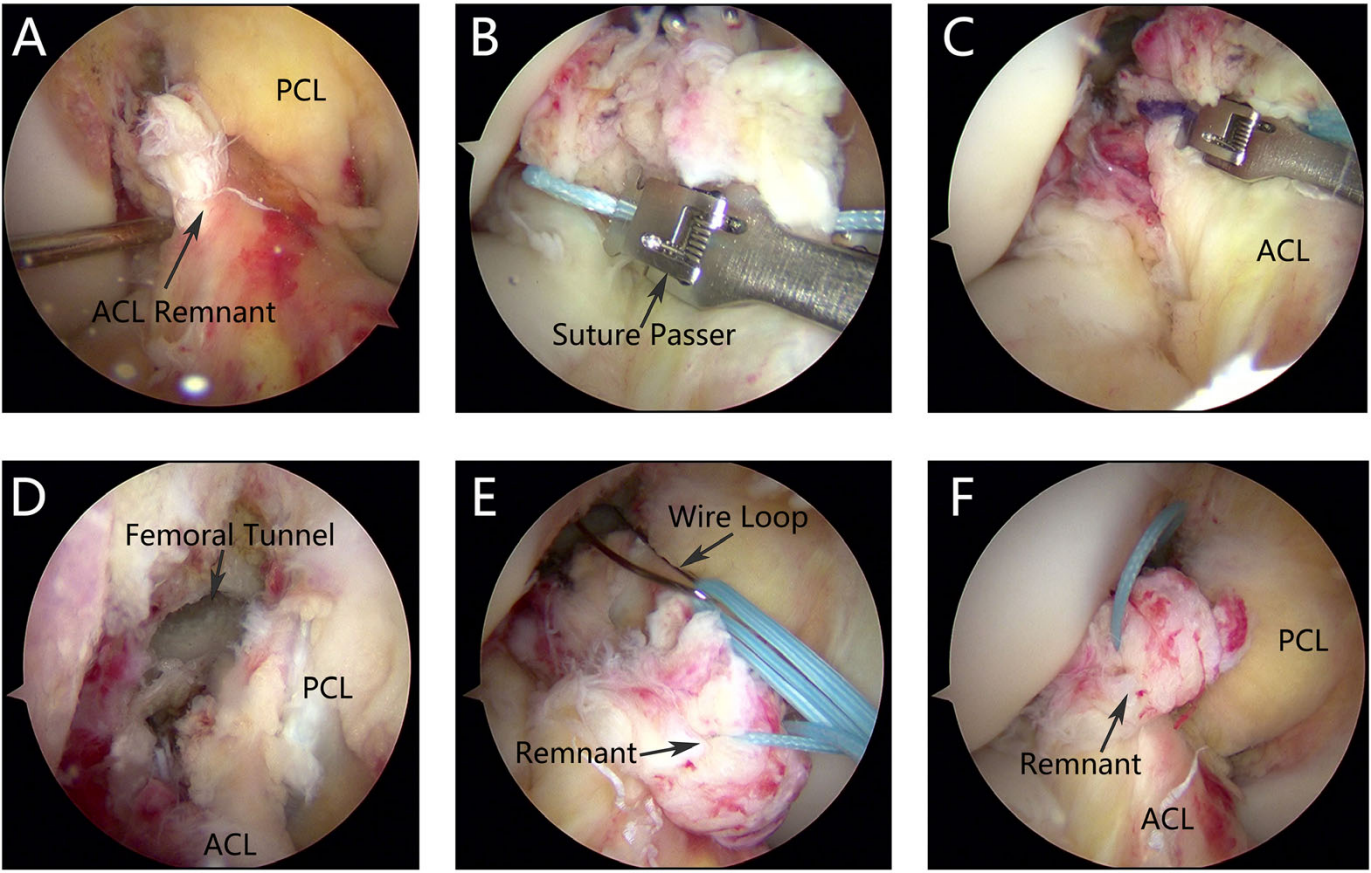
Arthroscopic views of the right knee obtained through the anterolateral portal. (A) Arthroscopic assessment showing rupture of the ACL with preservation of remnant fibers. (B) The ACL remnant is initially repaired using a cinch‐loop suture. (C) An additional encircling reinforcing stitch is passed through the remnant tissue to enhance circumferential capture. (D) Creation of the femoral tunnel using a transtibial approach. (E) The suture limbs are shuttled using a passing wire loop. (F) The suture limbs are subsequently pulled into the femoral tunnel in preparation for cofixation with the synthetic ligament. (ACL, anterior cruciate ligament; PCL, posterior cruciate ligament.)

Diagnostic arthroscopy is performed to evaluate the ACL remnant (Figure 2A), with particular attention to tissue quality and fiber continuity. Only unstable or impinging fibers are selectively debrided. The remnant surface is gently freshened using a shaver while preserving viable tissue.

### Step 2: Tunnel Creation

#### Tibial Tunnel

A tibial guide is positioned slightly posterior within the native ACL footprint. A guidewire is advanced, followed by reaming of a 7.5 mm tibial tunnel.

#### Femoral Tunnel

The femoral tunnel was created using a transtibial approach. Tunnel positioning aimed to achieve a nearly isometric graft behavior, guided by established anatomic landmarks and previously described isometric principles.^13^ A guidewire is advanced into the femur under arthroscopic visualization to achieve a near‐isometric graft position, and a 7.5 mm femoral tunnel is reamed accordingly (Figure 2D).

### Step 3: Sleeve‐Like Remnant Repair Using a Cinch‐Loop Configuration

**FIGURE 3:**
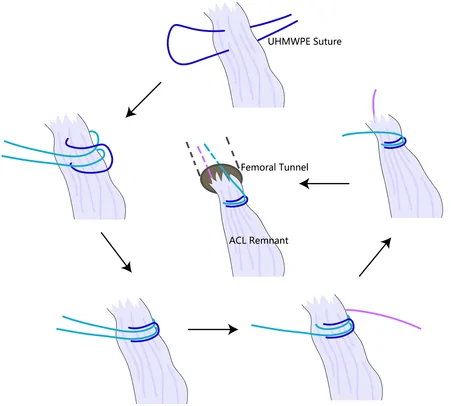
Arthroscopic schematic illustration showing the remnant repair technique. A cinch‐loop suture is used to capture the viable ACL remnant fibers, followed by an additional encircling reinforcing stitch passed through the existing loop and remnant tissue, creating a circumferential sleeve‐like construct over the synthetic ligament. (ACL, anterior cruciate ligament; UHMWPE, ultrahigh molecular weight polyethylene.)

Remnant repair is performed before graft passage to facilitate controlled coverage of the synthetic ligament. An Arthrex Scorpion suture passer loaded with a No. 2 ultrahigh molecular weight polyethylene suture (Arthrex, Naples, FL) is introduced through the accessory portal and passed through the ACL remnant (Figure 2B). The suture ends are retrieved and passed back through the loop to create a cinch‐loop configuration securing the remnant tissue. An additional 2 to 0 absorbable suture is then passed through the remnant and used as a shuttle to complete a circumferential encircling suture configuration (Figure 2C). This construct enables circumferential remnant capture and allows the remnant to form a sleeve‐like structure capable of enveloping the synthetic ligament (Figures 2E,F and 3).

### Step 4: Synthetic Graft Passage and Femoral Fixation

**FIGURE 4:**
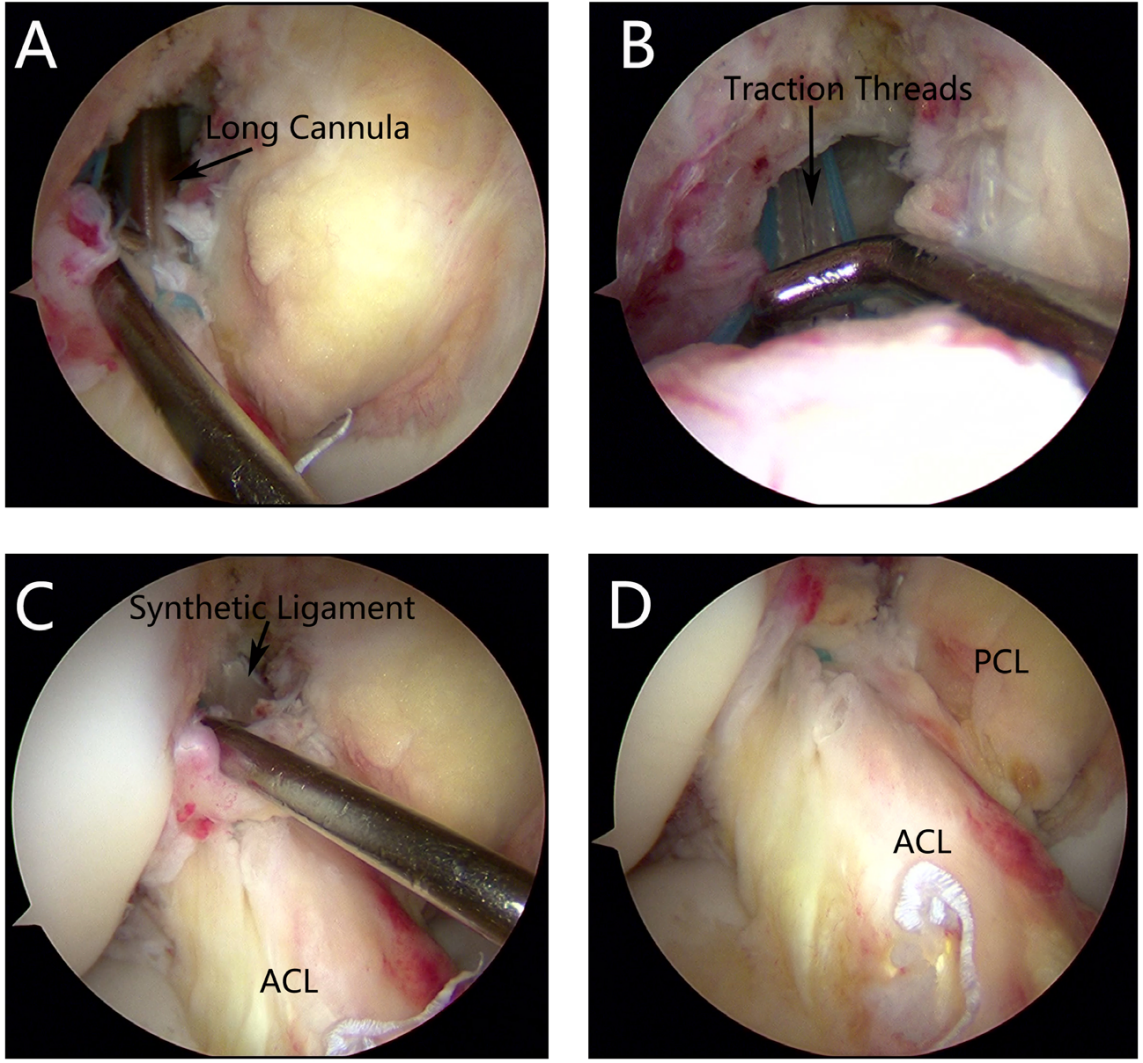
Arthroscopic views of the right knee obtained through the anterolateral portal. (A) Insertion of a long cannula into the femoral tunnel via the tibial tunnel. (B) The traction threads of the synthetic ligament are shuttled through the tibial tunnel, passed through the sleeve‐like repaired ACL remnant, and pulled into the femoral tunnel. (C) The synthetic ligament is advanced into the femoral tunnel, while the remnant repair suture limbs are intentionally left untensioned. (D) Final tensioning of the remnant repair sutures draws the repaired remnant proximally to cover the synthetic ligament. (ACL, anterior cruciate ligament; PCL, posterior cruciate ligament.)

The synthetic ligament (NGAL; Ligatech, Shanghai, China) is passed through the tibial tunnel and seated into the femoral tunnel under arthroscopic visualization (Figure 4A,B). The previously placed remnant sutures are tensioned to guide the remnant toward the femoral footprint and ensure that it drapes evenly around the graft (Figure 4C,D). Femoral fixation is achieved using a 8 mm × 25 mm titanium interference screw (Ligatech, Shanghai, China), securing the synthetic ligament and remnant sutures side‐by‐side within the femoral tunnel.

### Step 5: Tibial Fixation

After femoral fixation, the graft is tensioned distally while the knee is cycled to confirm isometry. With the knee positioned at 90° of flexion, tibial fixation is then performed using a second 8 mm × 25 mm titanium interference screw (Ligatech, Shanghai, China) to secure the synthetic graft.

### Step 6: Final Assessment and Closure

**FIGURE 5:**
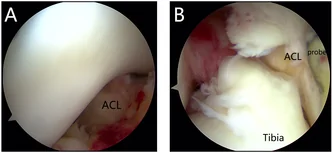
Arthroscopic views of the right knee obtained through the anterolateral portal during final assessment. (A) Full knee extension confirming absence of notch impingement. (B) Confirmation of complete containment of the synthetic ligament within the repaired remnant sleeve, without posterior escape or tibial‐side perforation. (ACL, anterior cruciate ligament.)

The knee is taken through a full range of motion to exclude notch impingement and extension loss (Figure 5A), while simultaneously confirming graft isometry between the synthetic ligament and the repaired remnant throughout flexion‐extension cycles. Arthroscopic inspection is then performed to confirm that the synthetic ligament is completely enveloped within the repaired remnant sleeve, with appropriate tension, and without posterior escape or perforation through the tibial‐side base of the remnant (Figure 5B).

## DISCUSSION

The hybrid technique described in this technical note addresses the inherent trade‐off between immediate mechanical stability and biological integration in ACL reconstruction. Rather than prioritizing a single reconstructive paradigm, this approach deliberately integrates synthetic ligament reconstruction with remnant repair to allow each component to function within its respective strengths through intraoperative design.

From a mechanical standpoint, the synthetic ligament provides immediate load‐bearing capacity and predictable tensile strength, which are particularly advantageous during early rehabilitation and in patients with high functional demands.^14^, ^15^ By sharing load with the repaired remnant, the synthetic graft reduces stress on the native tissue and may protect the remnant repair during the vulnerable early healing phase.^16^

Beyond mechanical support, preservation and repair of the ACL remnant offer important biological advantages.^17^, ^18^ Native remnant tissue contains vascular channels and neuromechanical receptors that may support biological integration and contribute to joint position sense and neuromuscular control.^18^, ^19^, ^20^ By configuring the remnant as a sleeve‐like structure enveloping the synthetic ligament, this technique aims to maximize preservation of these neuromechanical receptors, thereby maintaining the potential for proprioceptive signaling that synthetic materials alone cannot replicate. It should be recognized that the indications for isolated ACL repair remain limited, primarily involving proximal femoral‐sided tears with good tissue quality, such as modified Sherman type I or II lesions.^21^, ^22^ Recent studies suggest that suture tape‐augmented ACL repair can achieve short‐term outcomes comparable to standard reconstruction in selected patients.^23^ In the present hybrid technique, remnant repair is incorporated selectively based on intraoperative assessment rather than intended to broaden the indications for primary repair.

As a technical note, this report does not provide long‐term clinical or biomechanical outcome data, and prospective comparative studies with extended follow‐up are required to further evaluate the durability and clinical implications of this hybrid construct. The key pearls and pitfalls of the surgical technique are summarized in Table 1, and the principal advantages and disadvantages of this hybrid strategy are outlined in Table 2. In selected patients, this technique offers a reproducible integration of mechanical augmentation and biological preservation.

**TABLE 1:** Pearls and Pitfalls

Pearls	Pitfalls
Preserve viable ACL remnant tissue to create a sleeve covering the synthetic graft	Overaggressive remnant debridement compromises biological and neuromechanical benefits
Delay tightening remnant sutures until after graft passage	Premature suture tightening obstructs synthetic graft passage
Use a cinch‐loop suture followed by an additional encircling stitch to achieve circumferential remnant capture	Failure to adequately tension the repaired remnant may result in notch impingement or cyclops lesion formation
Place the tibial tunnel slightly posterior within the native ACL footprint	Excessively anterior tibial tunnel placement compromises remnant preservation
Use a transtibial femoral tunnel positioned in a nearly isometric zone	Nonisometric femoral tunnel placement increases graft‐tunnel motion
Cofix the synthetic graft and remnant on femoral side	
Confirm graft isometry and complete remnant containment dynamically	

ACL, anterior cruciate ligament.

**TABLE 2:** Advantages and Disadvantages

Advantages	Disadvantages
Provides immediate mechanical stability, facilitating early rehabilitation	Requires adequate remnant quality; severely attenuated or scarred remnants are not suitable
Allows load sharing between the synthetic graft and repaired remnant, potentially protecting the remnant during early healing	Precise tension management of the remnant repair is required to avoid impingement or cyclops lesion formation
Maximizes preservation of vascularized native tissue and neuromechanical receptors within the ACL remnant, maintaining the potential for biological integration and proprioceptive function	
Creates a sleeve‐like biological envelope that shields the synthetic ligament from direct intra‐articular exposure, potentially reducing synovial irritation	

ACL, anterior cruciate ligament.

## DISCLOSURES

The authors (P.W., Y.H., W.Y., Y.Z., W.Z.) declare that they have no known competing financial interests or personal relationships that could have appeared to influence the work reported in this paper.

## FUNDING

This work was supported by the Medical Science Research Project of Dalian (Grant No. DF2023006) and the Joint Fund of the Natural Science Foundation of Liaoning Province (Grant No. 2024‐MSLH‐107).
